# Association of Hashimoto’s thyroiditis and anti-thyroid antibodies with oral lichen planus: A cross-sectional study

**DOI:** 10.3389/fimmu.2022.967988

**Published:** 2022-08-16

**Authors:** Tianyu Zhang, Feifei Hou, Dan Liu, Hangfan Zhou, Yutong Sun, Xiaoting Deng, Yiming Xu, Yanxuan Xiao, Xianwen Wang, Chuanji Wu, Yang Meng, Peiyang Yuan, Xuemei Qiu, Lu Ye, Yuye Liang, Wei Wei, Lu Jiang

**Affiliations:** ^1^ State Key Laboratory of Oral Diseases, National Clinical Research Center for Oral Diseases, Department of Oral Medicine, West China Hospital of Stomatology, Sichuan University, Chengdu, China; ^2^ Department of Emergency, West China Hospital, Sichuan University, Chengdu, China

**Keywords:** oral lichen planus, Hashimoto’s thyroiditis, autoantibodies, TPOAb thyroperoxidase antibodies, TgAb thyroglobulin

## Abstract

Hashimoto’s thyroiditis (HT) and its autoantibodies may be associated with oral lichen planus (OLP). In this cross-sectional study, we aimed to assess the relationship among HT, auto-anti-thyroid antibodies, and OLP in a Chinese population of 247 patients with oral lichen planus. Clinical manifestations of OLP were evaluated using the Thongprasom scoring system and clinical type. The diagnosis of HT was based on thyroid function, anti-thyroid peroxidase antibody (anti-TPOAb) and anti-thyroglobulin antibody (anti-TgAb) detection, and ultrasonography. The prevalence of HT in all patients with OLP was 39.68% (98/247); the prevalence in females with OLP was 46.24% (86/186), which was higher than that in males with OLP 19.67% (12/61) (P < 0.01). The titers of the two HT autoantibodies in females with OLP were higher than those in males (P < 0.01). The clinical manifestations of OLP, regardless of being evaluated using the Thongprasom system or clinical type, were not significantly associated with HT development or TPOAb (P = 0.864) or TgAb titers (P = 0.745). In this population-based southern Chinese cohort, the prevalence of HT in patients with OLP, particularly in female patients with OLP, was significantly higher than that in the general population. Female patients had higher HT autoantibody titers than male patients. However, the clinical manifestations of OLP were not significantly correlated with either HT development or auto-anti-thyroid antibody levels. The findings could help further elucidate the factors involved in the relationship between oral lichen planus and Hashimoto’s thyroiditis.

## 1 Introduction

Oral lichen planus (OLP) is a chronic inflammatory autoimmune disease of the oral mucosa mediated by T cells (1). The associated morbidity rate is approximately 0.5%–2.0% of the general population (2), with a substantially higher rate in middle-aged and older women (3). However, a valid systematic review and meta-analysis in 2021 of 66 studies (involving 500 424 patients) reported a global estimate of OLP prevalence of 1.01%. Furthermore, from the age of 40 years, the incidence of OLP-related morbidity increases considerably and progressively, and there is no difference in prevalence between the sexes (4). The specific etiology and pathogenesis of OLP remain unclear. Risk factors such as mental status, immune function, endocrine function, infections (including hepatitis C virus infection), and some systemic diseases (hypertension, diabetes mellitus, dyslipidemia, thyroid disorders, and chronic liver disease) may play important roles in OLP development (5–8).

Hashimoto thyroiditis (HT), considered an autoimmune thyroid disease (TD) related to T cells (9), affects approximately 2% of the total population and continues to rise; women are more susceptible to HT (10). The pathogenesis of HT is not known, and its development depends on genetic susceptibility, epigenetic inheritance, and environmental factors (11).

The relationship between OLP and HT has been studied. The prevalence of HT in patients with OLP is considerably higher than that in the common population, suggesting a correlation between OLP and HT (12–15). The thyroid autoantibodies include anti-thyroid peroxidase (TPO), anti-thyroglobulin (Tg), and anti-thyroid-stimulating hormone receptor antibodies. The most common autoantibodies expressed in patients with HT are thyroid peroxidase antibodies (TPOAb) and thyroglobulin antibodies (TgAb). Studies have shown that anti-thyroid antibodies also play a part in extra-thyroid diseases, mainly through the localization, category, function, and duration of antigens (16). Circulating autoantibodies against HT are associated with other extra-thyroid autoimmune diseases such as rheumatoid arthritis, celiac disease, and type 1 diabetes mellitus (17, 18). Anti-TPO and anti-Tg antibodies lack specificity, resulting in occasional reports of their extra-thyroid effects (19). Circulating autoantibodies against HT have also been detected in patients with OLP, suggesting a correlation between the diseases (20).

No definite conclusion can be drawn from the findings of previous studies regarding the relationship between OLP and HT. Previous studies on OLP and HT had notable limitations and problems, such as diverse diagnostic criteria for HT and OLP among studies. Moreover, some studies did not explicitly include oral lichenoid lesions, which are related to several systemic or local factors such as specific drugs, dental material, and graft-versus-host disease. Additionally, there is a lack of analyses of the correlation between HT autoantibody levels and OLP. This cross-sectional study was designed to further investigate the correlation between HT and OLP in the Chinese Han population and study the relationship between autoantibody levels of HT and the clinical manifestations of OLP.

## 2 Materials and methods

### 2.1 Study design and ethics statement

This study was approved by the Ethics Committee of the West China Hospital of Stomatology, Sichuan University (WCHSIRB-D-2017-187). Two hundred and forty-seven patients with OLP were referred to the Department of Oral Medicine, West China Hospital of Stomatology from January 1, 2018 to May 1, 2021. All patients signed an informed consent form before the study. Patient demographic information, including age, sex, and medical and medication history, was recorded.

### 2.2 Participant selection

#### 2.2.1 Inclusion criteria

The study involved patients with OLP who were mainly diagnosed based on their history, clinical manifestations, and histopathological biopsy report according to the World Health Organization diagnostic criteria (21). The lesions in the buccal mucosa, lingual body, hard palate, soft palate, and gingiva were symmetrical on both sides; the lesions appeared as white and gray–white stripes with small papules. The pathological biopsy criteria included hyperkeratosis of the epithelium, liquefaction of basal cells, and dense infiltration of lymphocytes in the intrinsic layer.

#### 2.2.2 Exclusion criteria

The exclusion criteria were as follows: patients who (1) were diagnosed with other oral mucosal diseases; (2) had severe systemic diseases, tumors, and other autoimmune diseases that seriously affect the quality of life, such as psoriasis, Behçet’s disease, and bullous diseases; (3) received immune preparations within 3 months; (4) used certain drugs or amalgam fillers that may cause oral lichenoid lesions; (5) had organ transplantation; and (6) were pregnant or lactating.

### 2.3 Evaluation of OLP clinical manifestations

The Thongprasom scoring system and clinical type were used to assess the clinical manifestations of OLP as follows (22): 0 = no lesion, normal mucosa; 1= mild white striae only, no atrophic areas; 2 = white striae with atrophic areas < 1 cm^2^; 3 = white striae with atrophic areas > 1 cm^2^; 4 = white striae with erosive areas < 1 cm^2^; and 5 = white striae with erosive areas > 1 cm^2^. The clinical manifestations of OLP were described as reticular type, atrophic type, and erosion type, representing “1,” “2&3,” “4&5,” respectively.

### 2.4 HT diagnostic criteria

Currently, the diagnosis of HT is established by a combination of clinical signs, thyroid function (TSH, T3, T4, FT3, and FT4), serum antibodies against thyroid antigens (mainly anti-TPOAb and TgAb), and thyroid color Doppler examination (23). The serum antibodies against the thyroid were detected by the clinical laboratory at the West China Hospital of Sichuan University. The detection method is Electrochemiluminescence immunoassay (ECLI). Meanwhile, diffuse goiter, with a tough texture, especially with the enlargement of the pyramidal lobe of the isthmus, should be suspected of HT regardless of the changes in thyroid function. If the blood is positive for TPOAb or TgAb, HT can be diagnosed. Diagnosis was made for each patient by two independent experienced endocrinologists at the West China Hospital of Sichuan University.

### 2.5 Statistical analyses

The collected data were analyzed using SPSS software (version 23.0, IBM Corp., Armonk, NY, USA). The 95% confidence interval (CI) was used to compare thyroid disease and HT prevalence between patients with OLP and the general population. Comparison of the sex parameters of patients with OLP was performed using the χ^2^ test. Analysis of variance and rank-sum test were used to compare OLP scores with thyroid disease and the HT group with the control group. Statistical significance was set at P ≤ 0.05.

## 3 Results

### 3.1 Demographic information

Two hundred and forty-seven patients with OLP (61 males and 186 females; mean age 45.21 ± 12.72 years) were recruited. Sixty patients had a history of systemic diseases (except HT). The information is presented in **Table 1**.

**Table 1:** Demographics information of the 247 patients with OLP.

Characteristic	OLP (n = 247)
**Mean age (years)**	45.21 ± 12.72
**Sex**	
Male Female	61 (24.70%) 186 (75.30%)
**Other systemic diseases**	111 (44.94%)
Hypertension Diabetes Thyroid disorders except HT Others*	11 (4.45%) 5 (2.02%) 51 (20.65) 44 (17.82%)
**Medication history**	
Yes No	16 (6.48%) 231 (93.52%)

Data are presented as mean ± standard deviation unless specified otherwise.

*Others: included hepatitis, gallstone, gastritis, gastric ulcer, rectal cancer, nephritis, prostatitis, pulmonary cyst, fibroid, breast nodule, cervical carcinoma, anemia

OLP, oral lichen planus; HT, Hashimoto’s thyroiditis.

### 3.2 Prevalence of HT in patients with OLP

Among the 247 patients with OLP, the prevalence of HT was 39.68% (95% CI=33.58%-45.78%). The prevalence of HT was 46.24% in female patients with OLP, which was considerably higher than that in male patients with OLP (19.67%) (**Table 2**).

**Table 2:** HT prevalence and its autoantibodies in patients with OLP.

Characteristic	Male (n = 61)	Female (n = 186)	Total	*P*
**HT**				
With (%) Without (%)	12 (19.67) 49 (80.33)	86 (46.24) 100 (53.76)	98 149	0.000
**TPOAb**				
Positive (%) Negative (%)	12 (19.67) 49 (80.33)	73 (39.25) 113 (60.75)	65 182	0.005
**TgAb**				
Positive (%) Negative (%)	4 (6.56) 57 (93.44)	47 (25.27) 139 (74.73)	51 196	0.002

OLP, oral lichen planus; HT, Hashimoto’s thyroiditis; TPOAb, thyroperoxidase antibodies; TgAb, thyroglobulin antibodies.

### 3.3 Anti-thyroid antibodies in patients with OLP

**Figure 1:**
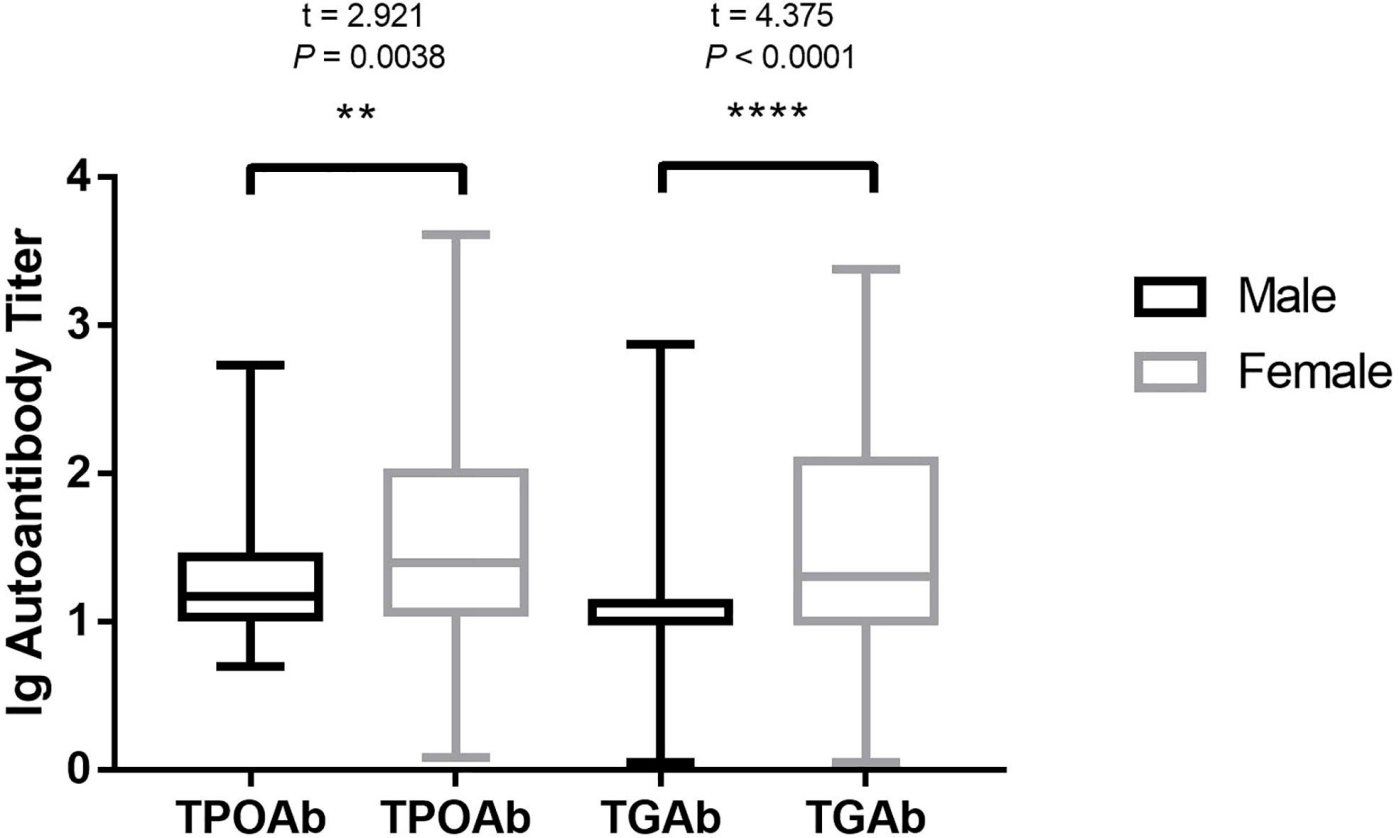
HT autoantibody in different sexes of patients with OLP. HT, Hashimoto’s thyroiditis; OLP, oral lichen planus; TPOAb, thyroid peroxidase antibodies; TgAb, thyroglobulin antibodies. **P < 0.01. ****P < 0.0001.

For the overall frequency of anti-thyroid antibodies, the positive rate of TPOAb in male patients with OLP was 19.67% (12/61) and that in female patients with OLP was 39.25% (73/186), which was considerably higher than that in male patients (P = 0.005). The positivity rate of TgAb in male patients with OLP was 6.56% (4/61) and that in female patients with OLP was 25.27% (47/186), which was considerably higher than that in male patients (P = 0.002) (**Table 2**). The titer of TPOAb base-10 logarithm in male patients with OLP was 1.23 and that in females was 1.52, which was considerably higher than that in male patients (t = 2.921, P = 0.0038). The titer of TgAb base-10 logarithm in male patients with OLP was 1.12 and that in females was 1.48, which was considerably higher than that in male patients (t = 4.375, P < 0.0001) (**Figure 1**).

### 3.4 Relations between OLP clinical manifestations and HT development

We divided the clinical manifestations in patients with OLP into scores 1–5 according to the clinical examination sign score; the prevalence of HT was 37.76%, 18.38%, 8.16%, 29.59%, and 6.11%, respectively. In the rank test, the P value was 0.877 > 0.05. Patients with OLP were divided into the following three types: reticular, atrophic, and erosion; the prevalence of HT was 37.76%, 26.53%, and 35.71%, respectively. In the rank test, the P value was 0.306 > 0.05. We statistically analyzed the HT detection rate in patients with OLP and clinical manifestations of OLP. The association between HT and OLP was independent of OLP clinical scores or subtypes. The results are presented in **Table 3**.

**Table 3:** Correlations between OLP clinical manifestations and developing HT.

Clinical manifestations	With HT (%)	Without HT (%)	Total	*P*
**OLP scoring**				0.877
1 2 3 4 5	37 (37.76) 18 (18.38) 8 (8.16) 29 (29.59) 6 (6.11)	69 (46.31) 17 (11.41) 12 (8.05) 36 (24.16) 15 (10.07)	106 35 20 65 21	
**OLP clinical type**				0.306
Reticular type Atrophic type Erosion type	37 (37.76) 26 (26.53) 35 (35.71)	69 (46.31) 29 (19.46) 51 (34.23)	106 55 86	

OLP, oral lichen planus; HT, Hashimoto’s thyroiditis.

OLP scoring: 0 = no lesion, normal mucosa; 1= mild white striae only, no atrophic areas; 2 = white striae with atrophic areas < 1 cm^2^; 3 = white striae with atrophic areas > 1 cm^2^; 4 = white striae with erosive areas < 1 cm^2^; and 5 = white striae with erosive areas > 1 cm^2^.

### 3.5 Relations between OLP clinical manifestations and HT autoantibodies

The clinical manifestations of OLP were divided into 1–5 points, and the Ig-TPO levels were 1.38, 1.63, 1.39, 1.53, and 1.45, respectively (rank test P = 0.864 > 0.05). The Ig-Tg levels were 1.37, 1.55, 1.48, 1.54 and 1.46, respectively (rank test P = 0.745 > 0.05). Patients with OLP were divided into the following three types: reticular, atrophic, and erosion. The Ig TPO levels were 1.38, 1.55, and 1.51, respectively (rank test P = 0.243 > 0.05). The Ig-Tg levels were 1.37, 1.53, and 1.52, respectively (rank test P = 0.142 > 0.05). We statistically analyzed the HT detection rate in patients with OLP and clinical manifestations of OLP. The HT antibody titer was not related to the OLP clinical scores or subtypes. The results are shown in **Table 4**.

**Table 4:** Correlations between OLP clinical manifestations and the HT autoantibody levels.

Clinical manifestation	Ig TPO level	*P*	Ig Tg level	*P*
**OLP scoring**		0.864		0.745
1 2 3 4 5	1.38 1.63 1.39 1.53 1.45		1.37 1.55 1.48 1.54 1.46	
**OLP clinical type**		0.243		0.142
Reticular type Atrophic type Erosion type	1.38 1.55 1.51		1.37 1.53 1.52	

OLP, oral lichen planus; HT, Hashimoto’s thyroiditis; TPO, thyroid peroxidase; Tg, thyroglobulin.

OLP scoring: 0 = no lesion, normal mucosa; 1= mild white striae only, no atrophic areas; 2 = white striae with atrophic areas < 1 cm^2^; 3 = white striae with atrophic areas > 1 cm^2^; 4 = white striae with erosive areas < 1 cm^2^; and 5 = white striae with erosive areas > 1 cm^2^.

## 4 Discussion

In this study, we aimed to explore the relationship between HT and OLP. The prevalence of HT in patients with OLP (39.68%) was significantly higher than that in the general population (2%) (10). The HT incidence rate in female patients with OLP was significantly higher than that in male patients. The titer of HT autoantibodies in female patients was considerably higher than that in male patients. However, HT prevalence and HT autoantibody levels in patients with OLP were not related to the OLP clinical manifestations.

Previous studies (20, 24) have inferred that OLP is related to HT, and this hypothesis has a theoretical basis. OLP and HT, as autoimmune diseases related to T-cell-mediated immune responses, seem to have some common immune triggers and pathogenic processes (25). Simultaneously, oral mucosal epithelial cells express antigens recognized by HT autoantibodies (TPOAb and TgAb) or antigens with structures similar to their recognized antigens (25). These might also be involved in the concurrent occurrence of these two diseases. Muzio (20) reported the HT prevalence rate in patients with OLP was 14.29% (15/105) in Italy. Tang (26) reported that the prevalence was 12.14% (71/585) in East China. Zhou (24) reported that the prevalence was 20.83% (40/192). These rates are significantly higher than the prevalence in the general population. Our results are consistent with the conclusions of previous studies: the prevalence of HT in patients with OLP (39.68%) was significantly higher than that in the general population. However, in this study, the prevalence of HT in patients with OLP was higher than that in previous studies, which may be related to the ethnic differences of patients and the OLP diagnostic criteria (oral lichenoid lesions were excluded).

Our study also implied the role of thyroid antibodies which may be associated with autoimmune diseases arising in the oral mucosa. The possible mechanisms related to HT and OLP may include the following two aspects. Patients with one specific autoimmune disease are more susceptible to the other (27). Autoimmune diseases may interact with each other, such as epitope spreading, and this may be one possible mechanism (28, 29). Patients with HT are more likely to suffer from other autoimmune diseases, such as OLP. Circulating thyroid antibodies may target oral/skin keratinocytes or cross-react with proteins on keratinocyte membranes, which stimulate cytotoxic T cells to release chemokines to promote the development of OLP lesions and attract more immune cells into developing lesions, eventually leading to more cryptic epitope exposure and OLP lesions (25). The active systemic immune state during the course of autoimmune diseases is also a possible precipitating factor. Previous studies (30, 31) have inferred that both OLP and HT are mainly Th1-type patterns of immune response. The levels of interferon gamma (IFN-γ) and interleukin-4 (IL-4) in the serum and lesions of patients with OLP are increased. Th1 cells may play a leading role in immune balance in the pathogenesis of OLP (30). Higher levels of IL-12 and IFN-γ and lower levels of IL-4 indicate that the Th1 immune response characterized by cellular immunity is the main type of HT (32). Furthermore, the TPOAb level was associated with an increased production of Th1 cytokines (31). An increase in systemic immune activity in patients with HT may lead to OLP susceptibility. A molecular simulation study on the pathogenesis of lichen and thyroid autoimmunity showed that human protein autoantigens involved in specific autoimmune diseases gave lichen and thyroid autoimmunity a certain genetic susceptibility (33).

Most previous studies on OLP and HT did not include the clinical manifestations of OLP or the level of HT autoantibodies in the correlation analysis of the two diseases. In our study, we discussed the relationship between OLP clinical manifestations and HT prevalence in patients with OLP for the first time. The results showed no definite correlations. We also studied the relationship between clinical manifestations of OLP and the level of HT autoantibodies. The results indicated that there was no definite correlation between the level of HT autoantibodies and clinical manifestations of OLP, and the same results were observed between the sexes. Based on a case–control study in 2017, Alikhani et al. (34) reported that the serum TPOAb level in patients with erosive OLP was higher than patients with non-erosive OLP, and that the blood levels of TPOAb were significantly correlated with an increased risk of erosive OLP. Possible reasons for these findings include ethnic differences, inclusion criteria for OLP (patients with oral lichenoid lesions were excluded), and sample capacity.

In our study, the positivity rate and serum HT autoantibodies levels in female patients with OLP were significantly higher than those in male patients. This is because females are susceptible to OLP and HT. The prevalence of autoimmune diseases is associated with age and sex (35). These findings may be related to an autoimmune response, female sex hormone levels and receptors, inactivation of chromosome X, and fetal microchimerism (36–38). Therefore, we speculate that the significant difference in the prevalence of HT in different sexes of patients with OLP may be related to predisposition of women with autoimmune disease. We believe that HT incidence in patients with OLP is related to sex, and that female patients with OLP are more likely to suffer from HT. Men represent one-tenth of HT prevalence rate in the general population (39). However, the gap narrows in patients with OLP. In our study, 46.24% of the females had HT, twice the prevalence in males (19.67%). The HT prevalence in male patients with OLP has significantly increased. The reason for this is unclear, and further clinical investigation is required.

Some aspects of the study need further improvement. This was a cross-sectional study and a causal relationship between the diseases could not be determined. Owing to the single-center nature and small number of patients, the samples may not have accurately represented the population; additionally, there was a lack of prospective studies. Moreover, potential confounding effects of changes in thyroid autoantibody levels were not excluded. Considering the cross-sectional design of this study, we could not directly examine or determine the potential mechanism underlying the association between OLP and HT. Large-sample multicenter prospective studies are needed to demonstrate our findings and search the potential pathophysiological mechanisms of these two diseases.

In conclusion, we found that OLP is associated with HT in the Chinese Han population. HT prevalence in patients with OLP was significantly higher than that in the general population; however, it was not significantly related to the OLP clinical manifestations or the level of HT autoantibodies. We speculate that the correlation between OLP and HT is mainly related to systemic immune status, epitope spreading, and other factors. Interestingly, women with OLP had a higher prevalence of HT and higher levels of thyroid autoantibodies. Moreover, HT prevalence in male patients with OLP has significantly increased, which needs to be investigated in future research. The findings could help further elucidate the factors involved in the relationship between oral lichen planus and Hashimoto’s thyroiditis.

## Data availability statement

The raw data supporting the conclusions of this article will be made available by the authors, without undue reservation.

## Ethics statement

The studies involving human participants were reviewed and approved by Ethics Committee of the West China Hospital of Stomatology, Sichuan University. The patients/participants provided their written informed consent to participate in this study.

## Author contributions

TZ: Conceptualization, methodology, investigation, data curation, formal analysis, writing – original draft preparation. FH: Conceptualization, methodology, investigation, data curation, formal analysis, writing – original draft preparation. DL, HZ, YS, XD, YMX: Investigation, data curation, formal analysis, writing – review and editing. YXX, XW, CW, YM, PY, XQ, LY, YL: Investigation, data curation, formal analysis. WW, LJ: Conceptualization, methodology, funding acquisition, project administration, supervision, validation, writing – review and editing. All authors contributed to the article and approved the submitted version.

## Funding

This work was supported by National Natural Science Foundation of China (81872208 and 82171809) and Sichuan Province Science and Technology Planning Project (2020JDJQ0013).

## Acknowledgments

We thank the individuals who participated in this study for their willingness to contribute to the advancement of science.

## Conflict of interest

The authors declare that the research was conducted in the absence of any commercial or financial relationships that could be construed as a potential conflict of interest.

## Publisher’s note

All claims expressed in this article are solely those of the authors and do not necessarily represent those of their affiliated organizations, or those of the publisher, the editors and the reviewers. Any product that may be evaluated in this article, or claim that may be made by its manufacturer, is not guaranteed or endorsed by the publisher.

## Abbreviations

HT, Hashimoto’s thyroiditis; IFN-γ, interferon gamma; IL, interleukin; OLP, oral lichen planus; Tg, thyroglobulin; TgAb, thyroglobulin antibody; TPO, thyroid peroxidase; TPOAb, thyroid peroxidase antibody
